# Copper Oxide Thin Films: Fabrication, Properties and Applications in Gas Sensing and Photoelectric Devices

**DOI:** 10.3390/ma19132918

**Published:** 2026-07-07

**Authors:** Anna Drabczyk, Paweł Uss, Wojciech Bulowski, Marta Mazur, Robert P. Socha

**Affiliations:** 1 CBRTP SA Research and Development Center of Technology for Industry, Zygmunta Modzelewskiego 77 St., 02-679 Warszawa, Poland; 2 Faculty of Non-Ferrous Metals, AGH University of Krakow, Mickiewicza 30 Av., 30-059 Kraków, Poland

**Keywords:** copper oxide thin films, CuO, Cu_2_O, atomic layer deposition (ALD), gas sensing, photoelectric devices, optoelectronic applications

## Abstract

Copper oxide (CuO) has emerged as a promising p-type semiconductor for a wide range of applications, including gas sensing and photoelectric devices. This is due to its narrow band gap, high chemical stability and low cost. In recent years, increasing attention has been paid to the development of high-quality CuO thin films with precisely controlled structural and electronic properties. Among various fabrication techniques, atomic layer deposition (ALD) provides unique advantages like excellent thickness control, conformality and tunability of film composition at the atomic scale. This review provides a comprehensive overview of CuO thin films with a particular focus on ALD-based fabrication approaches. First, conventional deposition methods are briefly discussed. Next, the fundamentals of ALD processes for CuO growth are presented including precursor chemistry, reaction mechanisms and the influence of key process parameters. Special attention is given to the correlation between deposition conditions and the resulting structural, optical and electrical properties of the films. Subsequently, the impact of these properties on device performance is analyzed in the context of gas sensing and photoelectric applications. Finally, current challenges and future perspectives are outlined, emphasizing the need for improved control over phase composition, defect engineering, and integration with nanostructured systems.

## 1. Introduction

Copper oxide-based materials, particularly cupric oxide (CuO) and cuprous oxide (Cu_2_O), have attracted significant attention as promising semiconductors for various applications, including gas sensing as well as photoelectric and optoelectronic devices. Their growing importance results from a unique combination of p-type semiconducting behavior, suitable optical band gaps, high chemical stability, relatively low cost and compatibility with numerous thin film fabrication techniques [1]. Depending on oxidation state and phase composition, copper oxides exhibit tunable structural, electrical and optical properties, which makes them attractive for a wide range of electronic, sensing and energy-related applications [2].

In recent years, increasing research interest has been focused on Cu_x_O_γ_ thin films dedicated to gas sensors, photodetectors, photovoltaic devices, photocatalytic systems and transparent electronics. In gas sensing, copper oxide-based materials have been investigated for the detection of gases such as CO_2_, NO_2_, H_2_S, NH_3_, CO, H_2_ and volatile organic compounds. Their sensing performance is strongly related to surface adsorption processes, defect concentration, grain boundaries and nanostructured morphology [3]. At the same time, copper oxides have also attracted attention in photoelectric and optoelectronic systems due to their visible light absorption, p-type conductivity and compatibility with heterojunction engineering. CuO and Cu_2_O thin films may be integrated with other metal oxides, silicon, transparent conductive oxides and carbon-based materials, enabling the development of photodetectors, transparent photoelectric devices and low-cost photovoltaic structures [4].

Among various metal oxide semiconductors, copper oxides are particularly attractive due to their relatively low cost, high natural abundance and lower toxicity compared to many conventional functional materials. Importantly, CuO and Cu_2_O belong to the group of earth-abundant materials, which makes them interesting from both economic and environmental perspectives. In contrast to systems relying on noble metals or complex compound semiconductors, copper oxide-based materials can be fabricated using relatively inexpensive precursors and scalable deposition methods. This gives them a favorable cost-to-performance ratio, especially in applications where low material consumption, device miniaturization and compatibility with industrial processing are required [5,6,7].

Copper oxide thin films can be deposited using numerous fabrication approaches, including sol–gel processing, spin coating, spray pyrolysis, chemical bath deposition (CBD), successive ionic layer adsorption and reaction (SILAR), sputtering, chemical vapor deposition (CVD) and atomic layer deposition (ALD). The selected deposition technique strongly affects phase composition, crystallinity, defect density, morphology, thickness and surface chemistry of the obtained films. Consequently, process optimization plays a critical role in tailoring the functional properties of Cu_x_O_γ_ layers toward specific applications. Particularly important is the ability to control phase evolution between Cu_2_O and CuO, since both materials exhibit substantially different band structures, conductivity mechanisms and adsorption behavior. Among the available deposition techniques, ALD has emerged as one of the most promising approaches for fabrication of high-quality Cu_x_O_γ_ thin films. This method enables excellent thickness control, high conformality and precise tuning of film composition at the atomic scale. These advantages are particularly important for nanostructured substrates, miniaturized devices and complex three-dimensional architectures. Therefore, ALD-based Cu_x_O_γ_ thin films are especially relevant for modern gas sensors, photodetectors and other integrated photoelectric devices.

This review provides a comprehensive overview of Cu_x_O_γ_ thin films with particular emphasis on their fabrication methods, physicochemical properties and potential applications in gas sensing and photoelectric devices. First, the crystal structure, defects and fundamental properties of Cu_x_O_γ_ systems are discussed. Subsequently, conventional solution-based and vapor-phase deposition techniques are reviewed, with special attention devoted to ALD processes. Finally, recent advances in sensing and photoelectric applications, together with economic perspectives and commercialization challenges, are summarized.

## 2. Structure, Defects and Physicochemical Properties of Cu_x_O_γ_ Thin Films

### 2.1. Crystal Structure and Phase Composition

Copper oxide thin films are mainly composed of two stable phases: Cu_2_O and CuO. These phases differ in crystal symmetry, oxidation state and electronic structure. Their coexistence or transformation strongly affects functional properties, especially in gas sensing and photoelectric devices. Cu_2_O crystallizes in a cubic cuprite structure, while CuO shows a monoclinic tenorite structure. These phases correspond to different oxidation states of copper: Cu^+^ in Cu_2_O and Cu^2+^ in CuO. Cu_2_O is characterized by a relatively open lattice and a direct band gap of approximately 2.0–2.2 eV. It is typically a p-type semiconductor due to the presence of copper vacancies. CuO exhibits a narrower band gap (≈1.2–1.7 eV) and a more distorted crystal structure, which influences carrier transport and defect states [8,9]. In thin films, intermediate or mixed phases may occur. Besides Cu_2_O and CuO, metastable phases such as Cu_4_O_3_ have also been reported, although they are less common. Phase coexistence is frequently observed, especially near transition conditions [10,11].

Recent studies emphasize that Cu_2_O/CuO heterostructures can improve functional performance. This is attributed to enhanced charge separation and interfacial effects, particularly in photoelectrochemical and sensing applications [12,13]. The phase composition of Cu_x_O_γ_ thin films is strongly dependent on oxygen availability, temperature and deposition method. Oxygen partial pressure is one of the most critical parameters. Lower oxygen partial pressure favors the formation of Cu_2_O. Under these conditions, Cu^+^ species are stabilized and oxidation remains incomplete. This behavior has been widely observed in sputtering, electrodeposition and low-temperature processes. Increasing oxygen partial pressure promotes the formation of CuO. The higher oxygen activity leads to further oxidation of Cu^+^ to Cu^2+^. This transition can also be induced by post-deposition annealing in air or oxygen-rich atmospheres [14]. Temperature plays a similar role. At moderate temperatures, Cu_2_O is often obtained, whereas higher temperatures promote conversion to CuO [15]. Studies on phase evolution show a gradual transition from Cu_2_O → Cu_4_O_3_ → CuO with increasing oxygen content or thermal energy. In practical systems, mixed-phase films are common. This is especially true when the process operates near the phase boundary. Such systems are not necessarily undesirable, as they may provide enhanced functional performance due to internal junction formation [16,17].

X-ray diffraction (XRD) is the primary technique used to determine crystal structure and phase composition of Cu_x_O_γ_ thin films. It enables identification of crystalline phases based on characteristic diffraction peaks. Cu_2_O exhibits diffraction peaks corresponding to cubic symmetry, typically indexed to (110), (111) and (200) planes. CuO shows distinct reflections associated with its monoclinic structure [18,19]. However, XRD analysis may be limited in thin films with low crystallinity or small grain size. Peak broadening and low signal intensity can complicate phase identification. This is particularly relevant for ALD-grown or nanostructured films. Therefore, complementary techniques are often required. Raman spectroscopy allows for clear distinction between Cu_2_O and CuO due to different vibrational modes [20]. X-ray photoelectron spectroscopy (XPS) provides information on the oxidation state of copper and enables detection of mixed-phase compositions [21,22].

Control over phase composition is critical for tailoring material properties. Cu_2_O is generally preferred for photoactive applications due to its wider band gap and favorable band alignment. CuO is often more suitable for gas sensing because of higher surface reactivity and stronger interaction with adsorbed species [23]. Importantly, mixed Cu_2_O/CuO systems can exhibit synergistic effects [24].

### 2.2. Defects and Stoichiometry

The properties of Cu_x_O_γ_ thin films are strongly affected by intrinsic defects and deviations from ideal stoichiometry. Both Cu_2_O and CuO are non-stoichiometric materials hence even small changes in the composition significantly influence various properties, including electrical, optical and surface-related ones [25,26].

In Cu_2_O, the dominant intrinsic defect is the copper vacancy (V_Cu). This defect acts as an acceptor center and is mainly responsible for the intrinsic p-type conductivity of this material. Copper vacancies are preferentially formed under oxygen-rich conditions wherein the oxidation process leads to the copper deficiency within the lattice [27]. As a result, Cu_2_O is commonly characterized as a metal-deficient semiconductor with composition close to Cu_2_−δO, where the concentration of copper vacancies directly determines hole concentration and electrical conductivity [28,29].

Other intrinsic defects may also occur in Cu_2_O, including oxygen vacancies (V_O) [30], copper interstitials, oxygen interstitials (O_i) and defect complexes. However, their formation energies are usually higher than those of copper vacancies [31,32,33]. Nevertheless, their concentration may increase during non-equilibrium deposition processes, especially during low-temperature synthesis or oxygen-deficient growth. These defects may introduce localized electronic states within the band gap and affect recombination processes, carrier transport as well as optical absorption [34,35]. Compared to Cu_2_O, CuO is characterized by a more complex defect chemistry. This results from its monoclinic crystal structure and strong electron correlation effects. Several intrinsic defects may coexist simultaneously. Their stability strongly depends on oxygen partial pressure, temperature and the position of the Fermi level. Consequently, CuO may exhibit broader variations in electrical conductivity and carrier concentration than Cu_2_O [36,37,38,39].

Non-stoichiometry in copper oxides is commonly described using compositions such as Cu_2_−δO or Cu_1_−δO, where δ represents deviation from the ideal atomic ratio. Even very small deviations may significantly modify the density of electronic states near the valence band. This directly influences carrier concentration, mobility, and transport mechanisms. In many cases, increased copper deficiency leads to higher hole concentration and enhanced p-type conductivity. Defects also play a critical role in determining optical properties. Localized defect states may act as trapping or recombination centers and influence absorption edge behavior, photoluminescence, and sub-band-gap optical transitions. Oxygen vacancies and defect complexes may additionally contribute to defect-related absorption bands and affect charge separation processes in photoactive systems [40,41].

## 3. Fabrication of Cu_x_O_y_ Thin Films

### 3.1. Conventional Solution-Based Methods

#### 3.1.1. Fabrication of Cu_x_O_y_ Thin Films via Spin Coating and Spray Pyrolysis

Conventional solution-based methods are widely used for the fabrication of Cu_x_O_γ_ thin films due to their simplicity, low cost and relatively easy scalability. These methods typically involve preparation of precursor solutions followed by film deposition and thermal treatment. Among the most commonly applied techniques are sol–gel processing, spin coating and spray pyrolysis.

Sol–gel-derived approaches are among the most commonly used solution-based techniques for Cu_x_O_γ_ thin film fabrication. This is due to their simplicity, low cost as well as relatively easy scalability. In these methods, sol–gel chemistry is combined with deposition techniques such as spin coating or dip coating. Such combination enables the transfer of the precursor solution onto the substrate surface by centrifugal spreading or immersion-based coating. Subsequent thermal treatment leads to solvent evaporation, precursor decomposition, crystallization and finally to the formation of Cu_x_O_γ_ thin films. The final film properties strongly depend on precursor chemistry, solvent composition, annealing temperature and deposition parameters. These approaches enable relatively simple fabrication of uniform Cu_x_O_γ_ thin films with controllable thickness, morphology, composition, and phase structure [42,43,44].

Several studies demonstrated that process parameters applied during sol–gel-derived deposition significantly influence the structural, optical, electrical and photoelectrochemical properties of the obtained films. For example, Aktar et al. reported solution-processed synthesis of Cu_x_O thin films fabricated by spin coating of CuI precursor solution dissolved in acetonitrile followed by NaOH-assisted conversion and thermal annealing. The study demonstrated that annealing temperature strongly influenced phase composition, crystallinity, morphology, optical properties, carrier density and photoelectrochemical performance of the obtained photocathodes. XRD and energy-dispersive X-ray spectroscopy (EDX) analyses confirmed formation of pure Cu_2_O at 250 °C, mixed Cu_2_O/CuO phase at 300 °C and pure monoclinic CuO at 350 °C. Thus, the annealing temperature strongly affected the phase composition of the obtained films. Next, as the annealing temperature increased, crystallite size also increased indicating improved crystallinity of the films. Analysis performed using scanning electron microscopy (SEM) demonstrated significant morphology evolution during annealing. The Cu_2_O film annealed at 250 °C exhibited grains of approximately 0.9 μm and visible void spaces. In contrast, the CuO film annealed at 350 °C showed smoother and more compact morphology with reduced grain boundaries and lower void density. In turn, optical analysis revealed that the indirect optical band gap of the CuO film annealed at 350 °C was approximately 1.3 eV. Mott–Schottky analysis confirmed p-type conductivity and revealed an increase in carrier concentration with increasing annealing temperature [45].

A similar approach was applied by Hashim et al. [46]. They investigated CuO thin films fabricated using a sol–gel spin coating technique on quartz substrates followed by annealing at 600 °C for 30 min. The study demonstrated that spin coating speed strongly affected film thickness as well as the optical and electrical properties of the deposited layers. An increase in the rotation speed from 1000 to 3000 rpm resulted in a significant reduction in film thickness from 215.43 to 39.97 nm. Simultaneously, the optical band gap decreased from 3.80 to 3.30 eV. The deposited CuO thin films exhibited Ohmic I–V behavior, while electrical conductivity decreased from 0.3584 to 0.0087 S m^−1^ with increasing spin coating speed. In contrast, resistivity increased from 2.79 to 114.57 Ω m. The authors attributed these changes mainly to variations in film thickness and carrier concentration induced by different spinning conditions.

Beyond conventional optimization of deposition parameters, recent studies also verified alternative functionalities and surface engineering strategies for sol–gel-derived Cu_x_O_γ_ thin films. These experiments included studies on wettability and hydrophobic behavior, biosensor-oriented CuO structures as well as annealing-induced modifications of optical and microstructural properties. For example, Bougharouat et al. [47] investigated the influence of annealing temperature on hydrophobic properties of CuO thin films fabricated by sol–gel spin coating. The films were annealed at temperatures ranging from 350 to 550 °C. Contact angle measurements demonstrated a significant increase in hydrophobicity with increasing annealing temperature with water contact angle increasing from 61° to 92°. In turn, XRD analysis confirmed improved crystallinity while studies performed using Fourier transform infrared (FTIR) spectroscopy revealed reduction of hydroxyl groups responsible for hydrophilic behavior. SEM observations additionally showed rougher and denser nanostructured surfaces after high-temperature annealing. In the next paper, Lillo-Ramiro et al. [48] studied CuO thin films synthesized by sol–gel spin coating with particular focus on optical and microstructural properties as well as potential biosensor-related applications. The authors investigated the influence of deposition speed, number of deposited layers and annealing treatment on film properties. XRD analysis confirmed monoclinic CuO structure while SEM observations revealed relatively homogeneous film morphology. Optical studies demonstrated band gap values ranging from 3.35 to 3.89 eV for as-deposited films and from 2.4 to 3.6 eV after annealing. Similar observations regarding the influence of annealing on optical and structural properties were reported by Al Fath et al. The authors demonstrated that annealing reduced structural disorder and defect density within the CuO films, which was reflected by lower Urbach energy values. Simultaneously, XRD analysis revealed improved crystallinity and increased crystallite size after annealing. Enhanced optical absorption in the visible range was also observed for annealed films, indicating potential applicability of the deposited CuO layers in photovoltaic systems [49].

The presented studies demonstrate that conventional solution-based approaches enable relatively precise control over the structural, optical, electrical and surface properties of Cu_x_O_γ_ thin films. At the same time, an increasing attention has recently been focused on environmentally friendly synthesis strategies based on biological and naturally derived compounds. For instance, Nwanna et al. [50] demonstrated an environmentally friendly approach for fabrication of CuO thin films using biosynthesis-assisted sol–gel spin coating. In this study, aqueous extract of *Allium cepa* peels was applied during preparation of the precursor solution. The deposited films were annealed at 400 °C for 3 h. XRD analysis confirmed formation of monoclinic CuO phase, while the estimated crystallite size was approximately 16.7 nm. SEM observations revealed spherical agglomerates of CuO nanoparticles with film thickness of approximately 106 nm. Optical studies additionally demonstrated strong absorption in the visible region and an optical band gap of approximately 1.48 eV, indicating potential applicability of the films in photovoltaic systems.

Another widely applied conventional technique for Cu_x_O_γ_ thin film fabrication is spray pyrolysis. Here, precursor solution is atomized into fine droplets and transported toward a heated substrate. Subsequently, the solvent evaporation and thermal decomposition lead to formation of the oxide layer. The properties of the obtained Cu_x_O_γ_ films strongly depend on such parameters as precursor composition, substrate temperature, spray rate, carrier gas flow and post-deposition annealing conditions [51,52,53,54].

Numerous studies demonstrated that optimization of these parameters significantly influences film crystallinity, morphology, thickness, optical properties and electrical behavior of the deposited layers. For example, Abdel-Galil et al. [55] fabricated CuO thin films using spray pyrolysis on glass and fluorine-doped tin oxide (FTO) substrates with particular focus on structural, optical, and electrochemical properties. The films were deposited at 400 °C using copper acetate precursor solution. SEM observations revealed homogeneous CuO layers composed of pyramidal-shaped crystalline grains with good adhesion to the substrate surface. XRD analysis confirmed formation of monoclinic CuO phase with nanostructured character of the deposited films. The authors additionally demonstrated high optical absorption and promising supercapacitive behavior of the CuO layers, indicating potential applicability in energy storage systems and optoelectronic devices.

In another paper, Diachenko et al. [56] investigated CuO thin films synthesized by pulsed spray pyrolysis with particular focus on structural and optical properties. XRD and Raman analyses confirmed formation of single-phase monoclinic CuO. Observations via SEM and atomic force microscopy (AFM) revealed rough nanostructured surfaces composed of grains with different shapes and sizes. The deposited films exhibited high absorption coefficients and optical band gap values within the range of 1.45–1.60 eV. Similar observations were reported by Kumar et al. [57], who investigated CuO thin films deposited by spray pyrolysis with particular focus on the influence of substrate temperature. Results of XRD analysis demonstrated a formation of monoclinic CuO phase with improved crystallinity at higher deposition temperatures. In turn, SEM observations revealed compact nanostructured morphology with relatively uniform grain distribution. A strong optical absorption in the visible range and band gap values characteristic of CuO semiconductors have also been observed thus indicating potential applicability of the films in optoelectronic and photovoltaic devices. A related study was presented by Hinna et al. [58], who investigated CuO thin films fabricated by spray pyrolysis for solar energy applications. The deposited films exhibited nanostructured morphology, good surface coverage, and monoclinic crystal structure. Optical studies revealed high absorption coefficients and suitable optical band gap values for absorber layer applications. The authors emphasized that spray pyrolysis enables relatively simple and scalable fabrication of CuO thin films with properties attractive for low-cost photovoltaic systems.

**Figure 1:**
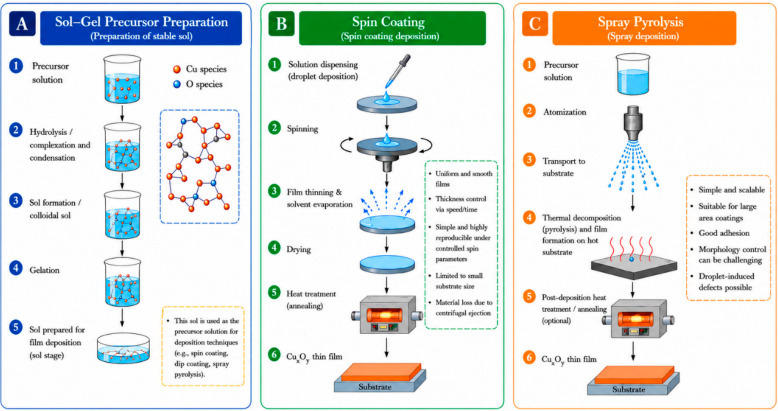
Schematic overview of selected conventional solution-based approaches for Cu_x_O_γ_ thin film fabrication. Panel (**A**) presents sol–gel precursor preparation, while panels (**B**) and (**C**) illustrate representative deposition routes based on spin coating and spray pyrolysis, respectively (this figure was generated with the assistance of ChatGPT Graphic Designer (GPT-5.5)).

The presented studies clearly demonstrate that conventional solution-based approaches enable relatively precise control over the structural, optical, electrical and surface properties of Cu_x_O_γ_ thin films. Depending on the selected deposition route and processing conditions, these methods make possible to fabricate films with tunable crystallinity, morphology, phase composition, thickness, wettability and optoelectronic performance. A schematic overview of the discussed approaches is presented in Figure 1. The scheme illustrates the general concept of sol–gel precursor preparation together with representative deposition routes based on spin coating and spray pyrolysis.

The discussed deposition techniques differ not only in the mechanism of film formation but also in achievable morphology, scalability, process control and thermal requirements. Spin coating is mainly used for fabrication of relatively uniform and smooth thin films with controllable thickness, whereas spray pyrolysis is particularly attractive for scalable and large-area deposition. A comparison of the main characteristics of these conventional deposition approaches is summarized in Table 1.

Overall, both spin coating and spray pyrolysis remain among the most attractive conventional techniques for Cu_x_O_γ_ thin film fabrication. Their popularity results from relatively low processing cost, simple experimental setup and broad possibility of tuning film properties through optimization of process conditions. Depending on the selected synthesis method, Cu_x_O_γ_ films with significantly different morphology, crystallinity, thickness and surface characteristics can be obtained. Despite certain limitations related to defect formation or morphology control, solution-based deposition methods begin to play an important role in development of low-cost Cu_x_O_γ_ thin film technologies.

#### 3.1.2. Chemical Bath Deposition (CBD)

Chemical bath deposition (CBD) is another widely investigated wet chemical method for fabrication of Cu_x_O_γ_ thin films. In this approach, the substrate is immersed in a precursor solution, where controlled chemical reactions lead to nucleation and gradual growth of the oxide layer directly on the substrate surface. Compared to spin coating or spray pyrolysis, CBD enables relatively low-temperature processing and does not require sophisticated vacuum equipment. The final properties of the deposited films strongly depend on precursor concentration, bath composition, pH, deposition time, temperature and post-deposition annealing conditions. Due to its simplicity, scalability and low fabrication cost, CBD has attracted considerable attention for preparation of Cu_x_O_γ_ thin films dedicated to sensing, photocatalytic, environmental and photovoltaic applications [59,60].

**Figure 2:**
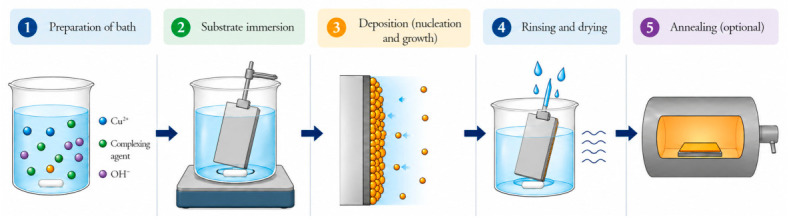
Schematic illustration of the chemical bath deposition (CBD) process for Cu_x_O_γ_ thin film fabrication (this figure was generated with the assistance of ChatGPT Graphic Designer (GPT-5.5)).

A simplified schematic representation of the CBD process applied for Cu_x_O_γ_ thin film fabrication is presented in Figure 2. The scheme illustrates immersion of the substrate in the precursor bath, nucleation and growth of copper oxide structures on the substrate surface, followed by rinsing, drying and thermal treatment leading to the formation of the final Cu_x_O_γ_ thin film.

Several studies demonstrated that CBD process parameters strongly affect the structural, optical, electrical and morphological properties of Cu_x_O_γ_ thin films. Sultana et al. [61] demonstrated that film thickness significantly influenced crystal quality, refractive index, dielectric constant and optical behavior of CuO layers deposited on silicon substrates. Here, it was observed that films with thicknesses of approximately 110 nm showed the most favorable overall properties, indicating the important role of controlled layer growth during the CBD process. XPS analysis additionally confirmed the oxidation states of copper and oxygen in the deposited films, supporting successful formation of CuO structures suitable for electronic and optical applications.

The influence of thermal treatment on CBD-derived Cu_x_O_γ_ systems was also widely investigated. Saadaldin et al. [62] demonstrated that annealing temperature strongly affected crystallization and surface morphology of copper oxide films prepared on glass substrates using an alternate immersion route. Increasing annealing temperature promoted structural ordering and modified the surface structure of the deposited layers. These observations confirmed that post-deposition thermal treatment played a crucial role in improving crystallinity and phase development of CBD-grown Cu_x_O_γ_ films.

Several studies additionally focused on nanostructured copper oxide layers obtained by CBD. Sadiq et al. [63] reported formation of polycrystalline monoclinic CuO films exhibiting rod-like nanostructured morphology. It was demonstrated that precursor concentration and annealing conditions affected crystallite size, preferred crystal orientation and optical band gap values. The optical band gap varied in the range of approximately 1.59–1.65 eV, indicating that CBD parameters can be utilized for tuning the optical response of CuO thin films.

Similarly, Lin et al. [64] investigated Cu_2_O thin films prepared by CBD and showed that precursor concentration significantly influenced film thickness, grain size and preferential crystal orientation. The preferential growth direction changed from the (200) to the (111) plane with increasing precursor concentration, demonstrating strong dependence of crystallization behavior on bath composition. Importantly, the authors emphasized that CBD enables relatively good step coverage and conformal coating of non-flat or microstructured substrates, which may be advantageous for fabrication of complex device architectures.

Overall, the reported studies confirm that CBD is a simple, low-cost and relatively low-temperature method for fabrication of Cu_x_O_γ_ thin films with tunable structural and functional properties. The method enables deposition over relatively large substrate areas without the need for complex vacuum equipment. Although the control of film uniformity and crystallinity is usually lower compared to vapor-phase techniques, CBD remains highly attractive for sensing, photocatalytic, environmental and photovoltaic applications due to its simplicity, scalability and easy processing conditions.

#### 3.1.3. Successive Ionic Layer Adsorption and Reaction (SILAR)

The successive ionic layer adsorption and reaction (SILAR) method is a solution-based deposition technique widely used for fabrication of metal oxide thin films, including Cu_x_O_γ_ materials. In contrast to conventional chemical bath deposition, the cationic and anionic precursor solutions are separated, and the substrate is sequentially immersed in individual solutions. A typical SILAR cycle consists of four steps: adsorption of ions on the substrate surface, rinsing to remove loosely bound species, reaction with oppositely charged ions, and final rinsing before the next cycle begins. Due to its layer-by-layer growth mechanism, SILAR enables relatively precise thickness control through adjustment of the number of deposition cycles. The method is considered attractive because of its low fabrication cost, simplicity, low processing temperature and compatibility with large-area substrates. Additionally, various process parameters such as precursor composition, pH, annealing conditions and number of SILAR cycles strongly influence the morphology, crystallinity and electrical properties of the deposited Cu_x_O_γ_ thin films [65,66].

Several studies demonstrated that SILAR-derived Cu_x_O_γ_ thin films may exhibit significantly different structural and functional properties depending on post-treatment conditions and precursor chemistry. For example, Lee and Wang [67] reported that Cu_2_O films prepared by SILAR and subsequently annealed in air gradually transformed into Cu_2_O–CuO mixed phases and finally monoclinic CuO, accompanied by band gap reduction from 1.90 to 1.34 eV and significant changes in carrier concentration and mobility due to heterojunction formation.

In another paper, Patwary et al. [68] emphasized that SILAR enables fabrication of stable, adherent and nanostructured Cu_x_O_γ_ films on various substrates at relatively low temperatures, while parameters such as precursor type, pH, annealing temperature and deposition cycles strongly affect grain morphology, crystallinity, optical band gap and electrical conductivity.

### 3.2. Vapor-Phase Deposition Techniques

Vapor-phase deposition techniques constitute an important group of methods used for fabrication of Cu_x_O_γ_ thin films showing enhanced crystallinity and improved adhesion. Importantly, these techniques allow for a relatively precise control over film thickness and composition. In contrast to solution-based approaches, vapor-phase methods rely on transport of material in the gaseous or vapor state, which is followed by condensation or chemical reaction on the substrate surface. Depending on the selected deposition route, vapor-phase methods enable fabrication of compact, dense, nanostructured or highly crystalline films with tunable optical, electrical and surface properties [69,70].

Among the most commonly investigated approaches, physical vapor deposition (PVD)- and chemical vapor deposition (CVD)-based techniques can be discussed.

#### 3.2.1. Physical Vapor Deposition (PVD) Technique

Physical vapor deposition (PVD) is a vacuum-based thin film deposition technique in which material is physically transferred from a solid target to the substrate surface through processes such as evaporation or sputtering. In sputtering-based PVD, energetic plasma ions bombard the target surface, leading to the ejection of atoms, ions or clusters that subsequently condense on the substrate and form a thin film. The composition, morphology, crystallinity and functional properties of the deposited layers strongly depend on parameters such as plasma power, working pressure, gas atmosphere, substrate temperature and deposition time [71]. Importantly, copper oxide-based thin films (Cu_x_O_γ_) can also be successfully fabricated using PVD methods. Two main sputtering strategies can be employed for the deposition of Cu_x_O_γ_ thin films. The first approach is based on reactive sputtering from a metallic Cu target in an oxygen-containing atmosphere, whereas the second utilizes a CuO target sputtered in inert Ar plasma.

**Figure 3:**
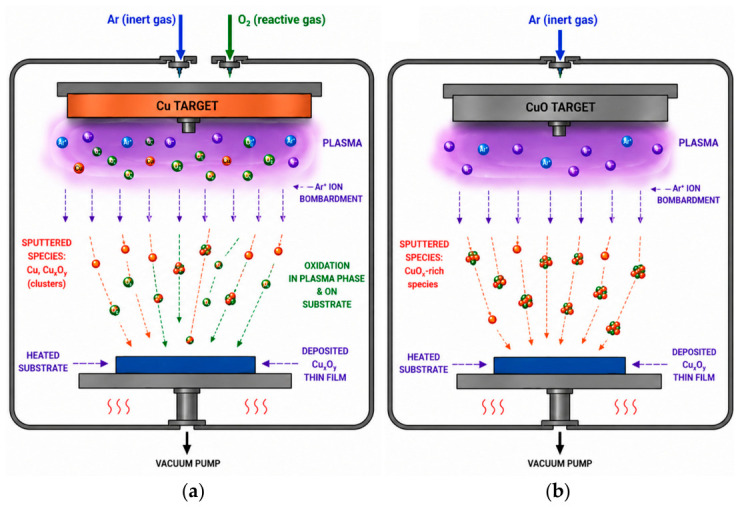
Schematic comparison of sputtering approaches for Cu_x_O_γ_ thin film deposition: (**a**) reactive sputtering from a metallic Cu target in Ar/O_2_ atmosphere; (**b**) sputtering from a CuO target in inert Ar atmosphere (this figure was generated with the assistance of ChatGPT Graphic Designer (GPT-5.5)).

A schematic comparison of both deposition routes is presented below in Figure 3.

Overall, numerous studies demonstrated that PVD-based approaches enable relatively precise control over phase composition, crystallinity, morphology, optical response and electrical behavior of Cu_x_O_γ_ thin films. Depending on deposition parameters and post-treatment conditions, these techniques allow for fabrication of various phases with properties tailored toward wide area of potential applications [72,73]. A comparison of representative PVD-based studies reported for Cu_x_O_γ_ thin films is summarized in Table 2.

The presented studies clearly demonstrate that PVD-based techniques enable broad tunability of structural, optical, electrical and electrochemical properties of Cu_x_O_γ_ thin films. Importantly, oxygen partial pressure during sputtering was identified as one of the most critical parameters controlling phase evolution between Cu, Cu_2_O, Cu_4_O_3_ and CuO. At the same time, the temperature of the substrate as well as post-deposition annealing strongly influenced crystallinity, grain growth, defect density and carrier transport behavior of the deposited films. Several studies additionally showed that even small modifications of sputtering conditions may significantly affect morphology and functional properties of Cu_x_O_γ_ layers. Depending on the applied deposition atmosphere and thermal treatment, the obtained films exhibited substantially different optical absorption, electrical conductivity, Hall mobility, capacitance and sensing response. Compared with conventional solution-based approaches, PVD techniques generally enable fabrication of denser and more uniform films with improved adhesion, reproducibility and thickness control.

#### 3.2.2. Chemical Vapor Deposition (CVD)

Chemical vapor deposition (CVD) is one of the most widely used vapor-phase techniques for the fabrication of high-quality thin films with controllable composition, crystallinity and morphology. Here, volatile precursors are transported in the gas phase into a heated reaction chamber, where they undergo thermal decomposition and/or chemical reactions near the substrate surface, leading to thin film formation. In the case of Cu_x_O_γ_ materials, the final phase composition strongly depends on precursor chemistry, substrate temperature, oxygen activity, pressure and gas flow conditions. By adjusting these parameters, different copper oxide phases, including Cu_2_O, Cu_4_O_3_ and CuO, can be obtained. Compared to many solution-based methods, CVD techniques generally enable improved film uniformity, conformality, adhesion and thickness control [83,84,85].

**Figure 4:**
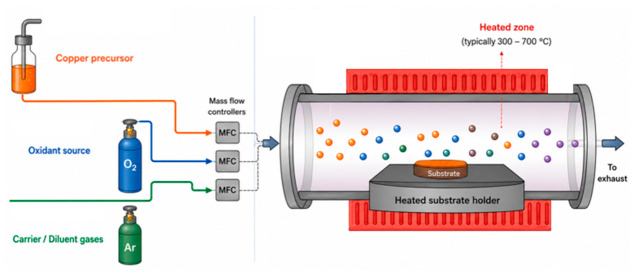
Simplified schematic illustration of chemical vapor deposition (CVD) process for Cu_x_O_γ_ thin film fabrication, including precursor delivery, gas-phase transport, thermal reactions and film growth on a heated substrate (this figure was generated with the assistance of ChatGPT Graphic Designer (GPT-5.5)).

A simplified schematic representation of the CVD process used for Cu_x_O_γ_ thin film fabrication is presented in Figure 4. The scheme illustrates introduction of the copper precursor, oxidant and carrier gas into the heated reactor chamber, followed by gas-phase transport, surface reactions and subsequent deposition of the Cu_x_O_γ_ thin film on the substrate surface.

Chemical vapor deposition techniques have been extensively investigated for the fabrication of Cu_x_O_γ_ thin films due to their ability to provide relatively high-purity materials, good thickness control and tunable structural properties. Numerous studies demonstrated that deposition temperature, oxygen partial pressure, precursor chemistry and reactor pressure strongly influence phase composition, crystallinity, morphology and electrical performance of the obtained films. Depending on the applied process conditions, CVD approaches enable formation of Cu_2_O, CuO or mixed-phase copper oxide structures with properties suitable for photovoltaic, sensing and catalytic applications.

For example, Eisermann et al. [86] investigated Cu_2_O thin films deposited by CVD on sapphire (0001) and MgO (100) substrates. The deposition was performed using copper(II) acetylacetonate as the copper precursor and oxygen as the oxidant source. The study demonstrated that oxygen flow strongly affected phase formation, enabling selective deposition of Cu_2_O or CuO. Low oxygen flow (20 sccm) resulted in Cu_2_O formation while higher oxygen flow (150 sccm) led to the growth of CuO. XRD analysis revealed preferential Cu_2_O (111)/(200) orientation on sapphire and epitaxial-like Cu_2_O (100) growth on MgO. This was due to the small lattice mismatch between Cu_2_O and MgO. In turn, SEM and AFM analyses demonstrated polycrystalline morphology with grain sizes reaching approximately 0.5–1.0 μm and surface roughness around 150 nm for films grown on MgO. Raman spectroscopy additionally indicated the presence of minor CuO inclusions within the Cu_2_O layers. Hall and thermoelectric measurements confirmed p-type conductivity with carrier concentrations in the high 10^15^–low 10^16^ cm^−3^ range and Hall mobilities of 5–8 cm^2^ V^−1^ s^−1^.

A different low-temperature CVD approach was reported by Chua et al. [87], who deposited high-purity Cu_2_O thin films using a copper(I) amidinate precursor and degassed water at substrate temperatures within the range 125–225 °C. The developed process enabled fabrication of pinhole-free single-phase Cu_2_O films with low contamination levels and controlled morphology. The study demonstrated also that the deposition temperature and pressure strongly influenced growth rate, crystallinity and electrical properties of developed materials. SEM observations revealed that lower deposition temperatures led to the fabrication of smooth and uniform films while higher temperatures contributed to the island growth and rough polycrystalline morphology. XPS and Raman analyses confirmed high phase purity without detectable CuO contamination in the bulk films. Hall measurements showed mobilities exceeding 14 cm^2^ V^−1^ s^−1^ for thicker films, while optical studies indicated tunable band gap values in the range of approximately 2.4–2.6 eV depending on film thickness.

Tian et al. [88] reported synthesis of CuO thin films using pulsed spray evaporation CVD (PSE-CVD) with copper acetylacetonate dissolved in ethanol as the precursor solution. The study demonstrated that deposition pressure and substrate temperature strongly affected growth kinetics and phase evolution. Pure monoclinic CuO was obtained at 300 °C, whereas lower temperatures led to Cu_2_O or mixed Cu_2_O/CuO phases. XRD analysis revealed nanocrystalline CuO films composed of dome-shaped loosely packed grains with average crystallite sizes of approximately 58–60 nm. Optical measurements indicated a direct band gap of approximately 1.81 eV. The deposited CuO films additionally exhibited good thermal stability up to approximately 450 °C and showed promising catalytic activity toward propene oxidation achieving complete conversion at significantly lower temperatures compared with non-coated substrates.

Besides conventional thermal CVD, several modified approaches have also been investigated for Cu_x_O_γ_ thin film fabrication, including mist CVD and aerosol-assisted chemical vapor deposition (AACVD). These techniques utilize liquid precursor solutions converted into aerosol or mist droplets that are subsequently transported into the heated reaction zone. Compared with conventional CVD, such approaches offer simplified precursor delivery, lower equipment cost and improved suitability for solution-processable precursors while still maintaining relatively good control over film composition and morphology.

For instance, a mist CVD was described by Narin et al. [89] who investigated temperature-controlled phase evolution of copper oxide thin films using copper(II) acetylacetonate Cu(acac)_2_ precursor solutions. The study demonstrated that growth temperature strongly affected phase formation between Cu_2_O and CuO. Films deposited at 300–350 °C exhibited pure cubic Cu_2_O phase, whereas deposition at 400–450 °C led to the fabrication of monoclinic CuO. AFM and SEM analyses revealed clear morphology evolution associated with the phase transition, while optical measurements showed band gap reduction from approximately 2.45 eV for Cu_2_O to 1.38 eV for CuO. Raman and low-temperature photoluminescence studies additionally confirmed differences in defect structure and crystal quality between the obtained phases. The authors emphasized that mist-CVD enables relatively simple and controllable phase engineering of copper oxide thin films through temperature adjustment.

In turn, an AACVD was proposed by Abdul-Hussein et al. [90]. In this process, aerosolized precursor droplets are transported into the reactor and decomposed near the heated substrate surface. The study demonstrated that AACVD enables preparation of relatively uniform copper oxide-based coatings with controllable composition and functional properties. Due to the aerosol-assisted precursor delivery, the process allows for the use of less volatile precursor systems compared with conventional CVD approaches while maintaining relatively good scalability and deposition uniformity. The obtained AgO–CuO films exhibited promising antibacterial activity together with tunable structural and optical properties.

#### 3.2.3. Atomic Layer Deposition (ALD)

Atomic layer deposition (ALD) has emerged as one of the most promising techniques for the fabrication of high-quality Cu_x_O_γ_ thin films due to its exceptional thickness control, excellent conformality and atomic-scale growth precision. ALD is a vapor-phase thin film deposition technique based on sequential, self-limiting surface reactions. Unlike conventional chemical vapor deposition (CVD), where precursors are introduced simultaneously and reactions may occur continuously both on the substrate surface and in the gas phase, ALD separates individual reactants into distinct pulses. Consequently, film growth is controlled by surface chemistry rather than gas-phase reactions, enabling highly uniform coatings even on complex three-dimensional and high-aspect-ratio structures. Depending on the selected process conditions, ALD enables the fabrication of Cu_2_O, CuO, or mixed-phase Cu_x_O_γ_ thin films with tunable morphology, crystallinity, defect concentration and functional properties [91,92,93].

A typical ALD cycle consists of four consecutive steps: precursor pulse, purge, co-reactant pulse and a second purge. During the first half-cycle, the precursor molecules are introduced into the reaction chamber and chemisorb on available reactive sites of the substrate surface. Once all accessible surface sites are occupied, further adsorption becomes energetically unfavorable, resulting in a self-limiting reaction. Excess precursor molecules and gaseous by-products are subsequently removed from the chamber by an inert carrier gas, typically nitrogen or argon. In the second half-cycle, a co-reactant is introduced to react with the adsorbed precursor species, forming the desired solid material while removing residual ligands. A final purge step eliminates remaining reaction products and prepares the surface for the next deposition cycle [92,94].

The self-limiting nature of ALD reactions represents the key distinction between ALD and conventional CVD processes. In CVD, the deposition rate is generally governed by precursor transport and reaction kinetics, often resulting in non-uniform growth and difficulties in controlling film thickness, especially on substrates with complex geometries. In contrast, ALD growth proceeds through sequential surface saturation reactions, providing exceptional thickness uniformity, high conformality and excellent reproducibility. These advantages are particularly important for copper oxide thin films intended for advanced applications such as gas sensors, thin film transistors, photodetectors and photovoltaic devices, where precise control of thickness, stoichiometry and surface morphology is required [95].

One of the most important parameters describing ALD processes is the ALD process window. This term refers to the temperature range in which the growth mechanism remains dominated by self-limiting surface reactions and the growth per cycle (GPC) remains nearly constant. Within this temperature interval, precursor adsorption, ligand removal and surface reactions proceed efficiently while maintaining the characteristic self-limiting behavior of ALD. Outside this range, deviations from ideal ALD growth may occur. At temperatures below the ALD window, precursor molecules may condense on the substrate surface instead of undergoing controlled chemisorption. Such condensation can lead to a non-uniform growth, uncontrolled increases in film thickness and elevated impurity concentrations due to incomplete ligand removal. Conversely, at excessively high temperatures, thermal decomposition of precursor molecules may occur before self-limiting adsorption is achieved. Under these conditions, the process gradually shifts toward CVD-like behavior, resulting in loss of atomic-level thickness control and deterioration of film uniformity. Therefore, careful optimization of deposition temperature is essential for obtaining high-quality copper oxide thin films with controlled phase composition and desirable physicochemical properties [96,97,98].

Another fundamental parameter used to characterize ALD processes is the growth per cycle (GPC), commonly expressed in angstroms per cycle (Å cycle^−1^). GPC represents the average thickness increment obtained during a single ALD cycle and serves as a direct indicator of deposition efficiency and surface reaction behavior. Under ideal ALD conditions, GPC remains relatively constant within the ALD process window due to the self-limiting nature of the surface reactions. The GPC value is influenced by several process parameters, including deposition temperature, precursor chemistry, pulse duration, purge time and substrate surface chemistry. Insufficient precursor pulse times may prevent complete saturation of reactive surface sites, leading to reduced growth rates. Conversely, extending the pulse duration beyond the saturation point generally produces little or no additional increase in film thickness, which is a characteristic feature of self-limiting ALD growth. Similarly, inadequate purge times may allow for precursor overlap in the gas phase, potentially causing parasitic CVD-like reactions. GPC measurements are widely used to evaluate process efficiency, identify saturation conditions and determine the optimal ALD operating window [99,100,101].

The deposition of copper oxide thin films by atomic layer deposition is governed by sequential surface reactions occurring between copper-containing precursor molecules and oxidizing co-reactants. Similar to other ALD processes, film growth proceeds through self-limiting reactions that enable precise control of film thickness, composition, and phase purity. However, copper oxide ALD is particularly challenging due to the complex redox chemistry of copper, which can exist in multiple oxidation states, primarily Cu^+^ and Cu^2+^, leading to the formation of Cu_2_O and CuO phases depending on deposition conditions. The growth mechanism typically begins with exposure of the substrate surface to a copper precursor. During this step, precursor molecules chemisorb onto reactive surface sites, most commonly hydroxyl (-OH) groups present on oxide surfaces. The adsorption process proceeds until all available reactive sites become occupied, resulting in surface saturation. Because the reaction is self-limiting, further precursor adsorption is prevented once saturation is reached, regardless of additional precursor exposure. Following precursor adsorption, excess precursor molecules and volatile reaction by-products are removed by an inert gas purge. This step is essential for preventing undesired gas-phase reactions between the copper precursor and the oxidizing co-reactant. In the subsequent half-cycle, an oxidizing agent such as H_2_O, O_3_, O_2_ plasma, or oxygen-containing radicals generated in plasma-enhanced ALD processes is introduced into the reactor. The oxidant reacts with the adsorbed precursor species, removing organic ligands and forming Cu–O bonds on the surface. A second purge step then removes gaseous reaction products and prepares the surface for the next deposition cycle. One of the key mechanisms responsible for copper oxide growth is the ligand exchange reaction. During precursor adsorption, the organic ligands attached to the copper center interact with reactive surface groups. Subsequent exposure to the oxidant removes the remaining ligands through oxidation reactions, producing volatile by-products such as CO_2_, H_2_O, and various organic fragments. The efficiency of ligand removal strongly affects impurity incorporation, carbon contamination, film density, and electrical properties [93,102].

The growth mechanism can be illustrated using one of the most frequently reported ALD systems based on copper(II) acetylacetonate [Cu(acac)_2_] and ozone. During the precursor pulse, Cu(acac)_2_ molecules adsorb onto hydroxyl-terminated surface sites through ligand exchange reactions. Subsequent ozone exposure oxidizes the remaining ligands and generates Cu–O bonds, while simultaneously regenerating reactive surface species necessary for continued film growth. Similar mechanisms have been reported for other copper precursors, although the reaction pathways and by-products may differ depending on ligand chemistry and oxidizing agent strength [103].

The phase composition of the deposited film is closely related to the surface reaction chemistry. Strong oxidizing agents such as ozone and oxygen plasma generally favor complete oxidation of copper species and promote the formation of CuO. In contrast, milder oxidants or carefully optimized process conditions may stabilize Cu^+^ species and facilitate the growth of Cu_2_O. Therefore, precursor selection, oxidant chemistry, deposition temperature, and plasma conditions collectively determine the final oxidation state of copper and consequently the structural, electrical, and optical properties of the deposited films [104]. Furthermore, the self-limiting nature of ALD reactions enables excellent conformality even on substrates with complex geometries and high aspect ratios. This characteristic is particularly advantageous for the fabrication of Cu_x_O_γ_-based gas sensors, photodetectors, thin film transistors, and photovoltaic devices, where precise thickness control and uniform coating of nanostructured surfaces are essential for achieving reproducible device performance.

The precursor chemistry plays a crucial role in determining deposition temperature, growth kinetics, impurity incorporation, and phase composition of ALD-grown copper oxide films. Representative copper precursors commonly employed for ALD of Cu_x_O_γ_ thin films, together with their main advantages and limitations, are summarized in Table 3.

The choice of copper precursor significantly influences growth kinetics, deposition temperature, growth per cycle, impurity incorporation and phase composition. In general, highly volatile Cu(I)-based precursors enable lower deposition temperatures and faster surface reactions, whereas Cu(II) β-diketonates offer superior thermal stability but often require elevated process temperatures. Consequently, precursor selection remains one of the most critical factors governing the quality of ALD-grown copper oxide thin films.

Numerous studies have recently focused on optimization of ALD processes for copper oxide thin films intended for sensing, photoelectric and energy-related applications. Representative ALD-based studies reported for Cu_x_O_γ_ thin films together with applied precursors, deposition conditions and selected physicochemical properties are summarized in Table 4.

The studies summarized in Table 3. demonstrate that ALD-based techniques enable precise control over the phase composition, crystallinity and morphology of Cu_x_O_γ_ thin films. This is possible via careful optimization of precursor chemistry, oxidizing agents and deposition parameters. Importantly, both thermal ALD and plasma-assisted approaches allow for deposition at relatively low temperatures while maintaining good film uniformity and conformality. The presented studies also confirm that ALD-grown Cu_x_O_γ_ thin films show promising optoelectronic and sensing-related properties, including photoconductivity and tunable electrical characteristics. Moreover, the possibility of selective formation of Cu, Cu_2_O and CuO phases highlights the versatility of ALD processes for tailoring material properties toward specific applications.

Compared with wet chemical methods, PVD and CVD techniques, ALD offers superior thickness control, excellent film uniformity and highly conformal coating of complex three-dimensional structures. Wet chemical methods are generally simple and cost-effective but often suffer from limited control over film thickness and uniformity. PVD techniques enable the deposition of high-purity films but step coverage on complex geometries may be limited. CVD provides good scalability and relatively high deposition rates, although precise thickness control can be more challenging than in ALD. Owing to its self-limiting growth mechanism, ALD is particularly attractive for gas sensing and photoelectric applications, where uniformity, reproducibility, and defect control are critical for device performance.

## 4. Application of Cu_x_O_y_ Thin Films

### 4.1. Gas Sensing Applications

Copper oxide thin films have attracted significant attention for gas sensing applications due to their semiconducting properties, relatively low fabrication cost, chemical stability and compatibility with numerous deposition techniques. Among copper oxides, CuO is particularly interesting because it is a p-type semiconductor with a narrow band gap and high surface reactivity, which enables efficient interaction with various gas molecules. In contrast, Cu_2_O usually exhibits a wider band gap and different adsorption behavior, while mixed Cu_2_O/CuO heterostructures may additionally improve sensing performance due to the formation of internal junctions and enhanced charge transfer processes. The relatively high catalytic activity of copper oxides also contributes to improved adsorption and surface reaction kinetics, which is particularly beneficial for low-concentration gas detection. Moreover, copper oxide thin films can operate at relatively low temperatures compared to many conventional metal oxide sensors, which may reduce power consumption and broaden their potential applications in portable and wearable sensing devices [115,116].

#### 4.1.1. Fundamentals of Gas Sensing Mechanism in Cu_x_O_γ_ Thin Films

The gas sensing performance of Cu_x_O_γ_ thin films is primarily governed by surface adsorption processes, charge transfer phenomena and the resulting modulation of electrical conductivity. Unlike many widely investigated metal oxide semiconductors such as ZnO, SnO_2_ and TiO_2_, which are typically n-type materials, copper oxides generally exhibit p-type conductivity. Consequently, the sensing behavior of CuO- and Cu_2_O-based sensors differs significantly from that of conventional n-type metal oxide sensors. Under ambient conditions, oxygen molecules from the surrounding atmosphere are adsorbed onto the surface of Cu_x_O_γ_ thin films. These molecules subsequently capture electrons from the semiconductor and become ionized oxygen species. Depending on the operating temperature, different oxygen species may dominate, including O_2_^−^, O^−^ and O^2−^. At relatively low temperatures, molecular O_2_^−^ species are typically predominant, whereas higher temperatures favor the formation of O^−^ and O^2−^ species. The electron extraction associated with oxygen adsorption modifies the charge distribution near the semiconductor surface. Since Cu_x_O_γ_ materials are p-type semiconductors, removal of electrons effectively increases the concentration of holes near the surface, resulting in the formation of a hole accumulation layer (HAL). This region possesses higher conductivity than the bulk material and plays a critical role in determining sensor response. When the sensor is exposed to reducing gases such as hydrogen (H_2_), ammonia (NH_3_), carbon monoxide (CO), hydrogen sulfide (H_2_S) or volatile organic compounds (VOCs), these molecules react with the adsorbed oxygen species. Such reactions release electrons back to the semiconductor surface. The returned electrons recombine with holes, decreasing the hole concentration within the accumulation layer and consequently increasing the electrical resistance of the sensor. In contrast, oxidizing gases such as nitrogen dioxide (NO_2_) tend to extract additional electrons from the semiconductor or promote further oxygen adsorption. This process increases hole concentration near the surface, leading to a decrease in sensor resistance. Therefore, the direction of resistance change depends strongly on the chemical nature of the target gas and the dominant surface reactions [117,118,119].

**Figure 5:**
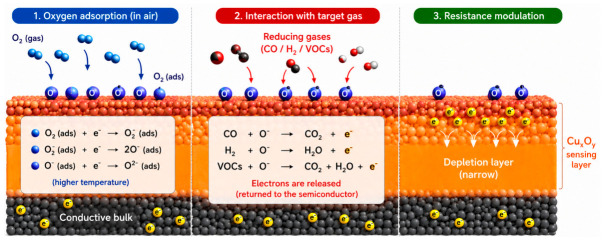
Generalized sensing mechanism of Cu_x_O_γ_-based chemiresistive gas sensors, including oxygen adsorption, interaction with reducing gases, electron exchange processes, and the resulting resistance modulation (this figure was generated with the assistance of ChatGPT Graphic Designer (GPT-5.5)).

The overall sensing mechanism is schematically illustrated in Figure 5.

Although the fundamental sensing mechanism remains similar, the actual sensor response may vary considerably depending on measurement conditions and material characteristics. For this reason, understanding the influence of operating parameters is essential for optimizing the performance of Cu_x_O_γ_-based gas sensing devices.

#### 4.1.2. Influence of Operating Conditions on Sensor Performance

The sensing response of Cu_x_O_γ_ thin films strongly depends on operating conditions, particularly temperature, humidity, gas concentration and exposure time. Among these factors, operating temperature is generally considered one of the most important parameters because it directly affects adsorption, desorption, diffusion, and surface reaction kinetics. At low temperatures, adsorption of oxygen and target gas molecules may be efficient. Nonetheless, reaction kinetics are often too slow to generate a significant sensing response. Increasing temperature enhances molecular mobility and accelerates surface reactions, resulting in improved sensitivity and shorter response times. However, excessively high temperatures may promote rapid desorption of adsorbed species, thereby reducing sensor response. Consequently, Cu_x_O_γ_-based sensors usually exhibit an optimum operating temperature at which the balance between adsorption and desorption processes is maximized [120,121].

Humidity represents another critical parameter influencing sensor performance. Water molecules may compete with oxygen and target gas molecules for active adsorption sites. In addition, hydroxyl groups formed on the sensor surface can alter charge transfer processes and modify the baseline resistance. As a result, high humidity frequently causes signal drift, reduced selectivity, and decreased reproducibility [122].

The dynamic behavior of the sensor is commonly described using response time and recovery time parameters. Response time corresponds to the period required to reach a specified fraction of the final signal after gas exposure, whereas recovery time describes the period necessary for the sensor to return toward its initial baseline after gas removal. These parameters are directly influenced by gas diffusion rates, adsorption strength, surface reaction kinetics, and material morphology [123,124].

#### 4.1.3. Structure–Property–Performance Relationships

The sensing performance of Cu_x_O_γ_ thin films is strongly governed by microstructural characteristics including grain size, porosity, crystallinity, surface roughness, defect density, and phase composition. Nanostructured films generally exhibit superior sensing performance compared with compact thin films due to their larger specific surface area and increased density of active adsorption sites. Smaller grains provide shorter charge transport pathways and increase the proportion of atoms located at surfaces and grain boundaries, where gas–solid interactions occur. Defects also play a crucial role in gas sensing. Oxygen vacancies, copper vacancies, grain boundaries, and other structural imperfections may serve as active adsorption centers and facilitate charge transfer processes. Consequently, controlled defect engineering has emerged as an effective strategy for enhancing sensor sensitivity. Phase composition represents another important factor. Cu_2_O and CuO exhibit different electronic structures, defect chemistries, and adsorption behaviors. Furthermore, mixed-phase Cu_2_O/CuO systems may form internal heterojunctions that facilitate charge separation and enhance sensing performance [125,126].

Numerous studies have demonstrated that increased porosity improves gas diffusion into the sensing layer, resulting in enhanced sensitivity and faster response/recovery characteristics [127,128,129]. Therefore, optimization of film morphology remains one of the key strategies for improving Cu_x_O_γ_-based gas sensors.

#### 4.1.4. Strategies for Sensitivity and Selectivity Enhancement

Although Cu_x_O_γ_ thin films exhibit promising sensing properties, further improvements in sensitivity, selectivity, stability, and detection limits are often required for practical applications. Hence several approaches have been investigated to enhance sensor performance. One widely employed strategy involves the introduction of dopants. Incorporation of elements such as Ag, Au, Pt, Pd, Zn, Ni, or Fe may modify carrier concentration, defect density, catalytic activity, and adsorption characteristics. Noble metal nanoparticles are particularly effective because they promote catalytic surface reactions and facilitate charge transfer processes [130,131].

Another important approach involves heterostructure engineering. Formation of p–n or p–p heterojunctions between Cu_x_O_γ_ and materials such as ZnO, SnO_2_, TiO_2_, WO_3_, or graphene can significantly enhance sensor response through improved charge separation and modulation of potential barriers at interfaces [132].

#### 4.1.5. Representative Cu_x_O_γ_-Based Gas Sensors

Following the discussion of sensing mechanisms and performance enhancement strategies, representative examples of Cu_x_O_γ_-based gas sensors reported in the literature are summarized in Table 5. The table compares target gases, operating temperatures, sensing responses, response and recovery times, detection limits, and key structural features of the investigated sensing materials.

As summarized in Table 5, the gas sensing performance of CuO-based thin films strongly depends on the deposition method, morphology, crystallinity, defect density and surface functionalization of the active layer. In most studies, porous nanostructured morphologies, reduced crystallite size and increased surface roughness were found to significantly enhance gas adsorption and charge transfer processes, leading to improved sensing response and selectivity. Additionally, incorporation of noble metals, heterostructure formation or elemental doping frequently resulted in enhanced sensing characteristics due to synergistic electronic and catalytic effects. Among the analyzed target gases, particularly high sensing responses were reported for H_2_S and NO_2_ detection, which is mainly associated with the strong interaction of these gases with CuO-based surfaces. Importantly, the reviewed studies demonstrate that CuO thin films can be successfully fabricated using a wide range of techniques, including PVD, CVD, spin coating, SILAR, spray pyrolysis and inkjet printing, enabling flexible optimization of structural and sensing properties for specific applications.

Another promising direction for the development of Cu_x_O_γ_-based gas sensors is the integration of artificial intelligence (AI) and machine learning (ML) approaches. Modern gas sensors often generate large amounts of data that may be influenced by temperature, humidity, sensor drift and cross-sensitivity toward multiple gases. AI-based algorithms can be employed to process these complex datasets, improve signal interpretation and enhance sensor selectivity and accuracy. Machine learning models may be trained to recognize characteristic response patterns associated with specific gases, enabling reliable detection even in complex gas mixtures. In addition to data analysis, AI may also support sensor design and material optimization. By combining experimental results with predictive models, machine learning can identify relationships between deposition parameters, material properties, defect concentration, morphology and sensing performance. Such approaches may significantly reduce the number of required experiments and accelerate the development of optimized sensing materials. Furthermore, AI-assisted systems can enable adaptive sensor operation, real-time calibration, predictive maintenance and integration into Internet of Things (IoT) platforms [148,149,150]. Therefore, the combination of Cu_x_O_γ_-based sensing materials with artificial intelligence is expected to become an important research direction for the next generation of high-performance gas sensors.

### 4.2. Photoelectric/Optoelectronic Applications

Copper oxide-based thin films and nanostructures have attracted considerable interest in photoelectric and optoelectronic applications due to their favorable semiconducting properties, tunable band gap, high optical absorption coefficient, and compatibility with numerous deposition techniques. Depending on the oxidation state and material stoichiometry, Cu_x_O_γ_ structures may exhibit different electrical and optical characteristics, enabling their application in photovoltaic systems, photodetectors, photoelectrochemical devices, and light-assisted sensing platforms. In particular, CuO and Cu_2_O materials are widely investigated because of their visible light activity, relatively low fabrication cost, and potential integration with other semiconductor materials in heterojunction-based structures.

Representative examples of Cu_x_O_γ_-based photoelectric and optoelectronic applications reported in the literature are summarized in Table 6.

As shown in Table 6, Cu_x_O_y_ materials demonstrate broad applicability in photoelectric and optoelectronic systems due to their tunable band gap, high optical absorption and favorable semiconducting properties. Depending on the oxidation state, stoichiometry and morphology, copper oxide structures can operate in photovoltaic, photocatalytic, sensing and photoelectrochemical devices. In many cases, nanostructuring and heterojunction engineering additionally improve charge separation efficiency and photoresponse. Consequently, Cu_x_O_y_ thin films remain promising candidates for low-cost and scalable optoelectronic technologies.

## 5. Market Perspectives and Economic Aspects of Cu_x_O_y_ Thin Films

### 5.1. Market Size, Demand and Sector Applications

The gas sensors market is in a dynamic growth phase. The global gas sensors market is projected to grow by $1.09 billion between 2026 and 2030, at a Compound Annual Growth Rate (CAGR) of 11.1%, and the gas detectors market is projected to reach $6.8 billion by 2031 [160,161].

The economic potential of copper oxides should be assessed through the lens of market segments where low unit price, rapid response, the possibility of production automation, and compatibility with mass-market systems are key. The most important application areas for gas sensors include air quality monitoring; industrial safety; Heating, Ventilation, and Air Conditioning (HVAC) systems; smart building systems; automotive; and smart home appliances. The review indicates that the growth in demand is primarily driven by environmental regulations, rising safety standards, and the development of connected devices [162].

In the area of CO_2_ and related gas detection, copper oxides are particularly interesting for applications where the highest absolute selectivity is not required, but stability, fast response time, and the ability to operate in a compact design are important. This applies, for example, to indoor air quality monitoring, leak detection in food packaging systems, monitoring fermentation processes, or assessing environmental conditions in airtight buildings. In these areas, implementation and operating costs often outweigh laboratory-record sensitivity parameters.

In photoelectrics, the market is more competitive, but at the same time offers a wider variety of niches. CuO and Cu_2_O are used in visible and near-infrared photodetectors, self-powered systems, imaging sensors, and transparent components. Publications in recent years indicate that hybrid CuO/rGO systems and photoelectrochemical (PEC)-type photodetectors with Cu_2_O(CuO) composites achieve good performance at a relatively low cost. From an economic perspective, it is important that such systems can be developed without the need for expensive photosensitive materials, making them competitive in applications where price and simplicity of integration are as important as high responsiveness [162,163].

### 5.2. Material and Production Costs

The greatest economic advantage is the ability to produce thin layers using scalable methods: sputtering, sol–gel, electrodeposition, spray pyrolysis, screen printing, or ALD. This lowers the cost of entry into production because it does not require expensive raw materials or complex, high-end lithography [112]. Copper is a widely available element, well-established in global supply chains, and relatively price-stable compared to precious metals and specialized materials. From an industrial perspective, this means less exposure to price risk and better predictability of production costs.

The material cost of CuO/Cu_2_O is typically lower than for sensors based on precious metals, halide perovskites, or 2D materials with a more complex supply chain. Furthermore, thin films use very little active material, so the bill of materials (BOM) cost per unit can be advantageous even with the addition of additional catalysts or heterojunction layers [164]. In practice, CuO and Cu_2_O are particularly interesting as active layers deposited on inexpensive ceramic, glass, or silicon substrates. In the case of photodetectors and photoconductors, a thin layer of copper oxide can be obtained by a simple chemical process and then combined with other materials, such as rGO, Si, or other oxides. Examples from the literature show that hybrid CuO/rGO or Bi:CuO/n-Si structures enable improved optical and electrical parameters while maintaining low manufacturing complexity. This is important because the simplicity of the process is a key source of cost advantage.

In gas sensors, the total device cost depends not only on the active layer itself, but also on heating, electronics, and packaging. In this context, it is important to note that this technology is not “absolutely cheap,” but rather “functionally cheap.” This means that the economic value stems from a favorable relationship between manufacturing cost and the achieved performance parameters such as sensitivity, responsiveness, operating range, miniaturization potential, and compatibility with electronics. If a CuO device requires higher energy consumption for heating, this may partially reduce its cost advantage, but with the use of micro-hotplates and local heating, this cost remains acceptable for many IoT applications.

CuO’s cost-effectiveness stems from three elements. First, it allows for the construction of very small sensors, reducing the cost of integration in IoT systems and portable devices. Second, it provides fast response and good sensitivity, reducing the cost of maintaining quality and increasing product usability. Third, it enables thin film production over a large area, supporting economies of scale. In photoelectrics, a similar logic applies even more strongly: CuO is described as a high-performance, low-cost material for visible light detection, and demonstrations on 4-inch wafers show that the process can be compatible with industrial-scale production. This is important because in this class of devices, success depends not only on the parameters but also on the feasibility of mass production on standard semiconductor equipment.

### 5.3. Competitive Advantages

Compared to NDIR, CuO-based sensors are smaller, cheaper to integrate, and more suitable for ultra-compact electronics, although they are inferior in selectivity and interference immunity. The greatest technological advantage of CuO and Cu_2_O is the ability to create p-n heterojunctions and hybrid structures, where a small change in composition or morphology can significantly improve device properties. In gas sensors, such systems increase charge transfer efficiency and amplify the gas response signal. In photodetectors, they improve carrier separation, limit recombination, and can increase sensitivity to specific spectral ranges. This design flexibility provides an advantage over simple single-phase materials.

Compared to noble materials, CuO offers significantly better raw material economy. In contrast, compared to organic and hybrid materials, it offers higher mechanical stability and better thermal resistance, although it often requires higher operating temperatures and more precise humidity control. In gas sensors, CuO also offers an advantage over many alternative semiconductor materials because it can act as a p-type oxide and easily form complementary systems with n-type oxides such as ZnO or SnO_2_. This leads to enhanced gas response via the junction effect. In optoelectronics, CuO exhibits an energy band favorable for visible light absorption, making it an interesting material for low-cost optical detectors.

It is worth noting that some publications point to the excellent performance achieved by CuO-based devices, even with simple fabrication methods. For example, a CuO/rGO hybrid photodetector demonstrated a broad response band from 395 to 945 nm, while a Cu_2_O(CuO) system operating without external bias achieved high responsivity and fast signal rise and decay times. These results are economically significant, demonstrating that it is possible to create devices of acceptable or high performance without costly epitaxial structures.

In photoelectricity, CuO and Cu_2_O compete with silicon, other metal oxides, perovskites, and TMDs. Their advantages lie in low material cost, compatibility with simple processes, and the potential for energy-sufficient devices. However, their weaknesses include lower carrier mobility, greater parameter variability, and the need for improved grain boundary engineering.

### 5.4. Major Barriers and Challenges

The biggest economic problem is not the cost of CuO itself, but rather the cost of stabilizing device parameters and bringing the technology to industrial repeatability. Without this, the material’s cost advantage can be eroded by low efficiency or excessive variation in production parameters. The review highlights limitations in gas sensors, with key challenges including low selectivity, humidity compensation, temperature-dependent response, signal drift, and active layer aging. From an economic perspective, such limitations increase the cost of calibration, testing, and servicing, thus increasing the total cost of ownership. In commercial applications, these elements can determine the total cost of ownership more than the price of the active material.

In photoelectric devices, however, challenges include microstructure control, band gap reproducibility, interface stability, and reducing recombination losses. The review on CuO-based sensors clearly emphasizes that many systems only perform well under specific temperature and humidity conditions, with some configurations requiring operating temperatures of several hundred degrees Celsius. Although microheaters reduce energy consumption, this remains a barrier in ultra-low-power applications. For the mass market, this means a compromise between performance and operating costs.

In photoelectric devices, implementation costs stem primarily from the need to control microstructure, interfacial purity, and crystalline defects. In many cases, increasing efficiency requires doping, creating heterostructures, or combining them with other conductive materials, which improves performance but increases process complexity. Economically, this means that CuO is especially attractive where moderately advanced but still inexpensive production technology can be employed. Furthermore, defect and interface control, as well as parameter stability over long-term operation, remain challenges in photoelectric devices. Doping can improve results, but it also increases process complexity. Consequently, the material’s cost advantage may be undermined by the costs of quality control and supporting processes. Therefore, the most promising variants offer improved performance while minimizing the increase in production complexity.

### 5.5. Economic Conclusions

Copper oxides in thin films represent a technology with high economic attractiveness and solid implementation potential, boasting a high cost/performance ratio. This means that with moderate development costs, a product that will satisfy many market segments can be achieved, even if it does not lead in absolute laboratory parameters. Their advantages lie in inexpensive raw materials, low material costs per device, compatibility with simple manufacturing methods, and the ability to integrate with modern sensor and photoelectronic platforms. This logic is particularly beneficial in high-volume sectors where reliability, low BOM costs, and production scalability are key.

For gas sensors, the most promising applications are in smart buildings, industrial safety, environmental monitoring, and diagnostic systems. In gas sensors, CuO makes the most sense in segments where miniaturization, low cost, and rapid response are key, rather than perfect single-gas selectivity.

In the case of photoelectrics, the prospects are linked to low-cost visible photodetectors, self-powered systems, and integrated multifunctional sensors.

The common denominator for both areas is that CuO does not have to replace all competing technologies to be cost-effective—it just needs to offer a better compromise between price, performance, and ease of production in selected segments.

At the same time, it should be clearly stated that the commercial success of this technology depends not on the material itself, but on solving problems related to selectivity, humidity, stability, and process control. If these barriers are reduced through heterostructures, doping, and mature thin film engineering, CuO and Cu_2_O could become among the most cost-effective materials for the low-cost gas sensor segment and selected classes of photodetectors. Ultimately, copper oxides should not be considered competitors to the best premium technologies, but rather as a solution with high economic utility, particularly in mass-produced and semi-mass-produced products.

## 6. Conclusions and Future Perspectives

Copper oxide thin films have attracted considerable attention due to their favorable semiconducting properties, low cost, earth abundance and broad applicability in gas sensing, photoelectric, and optoelectronic devices. As demonstrated throughout this review, the properties of Cu_x_O_γ_ thin films strongly depend on phase composition, defect structure, morphology, and deposition conditions. Various fabrication techniques, including solution-based methods, PVD, CVD and ALD, offer different advantages in terms of process complexity, scalability, film quality and control over material properties. Among them, ALD has emerged as a particularly promising approach due to its excellent thickness control, conformality and ability to produce highly uniform films.

Despite significant progress, several challenges remain. Precise control of phase composition between Cu_2_O and CuO, defect engineering, long-term stability and reproducibility continue to limit the practical implementation of Cu_x_O_γ_-based devices. In particular, a deeper understanding of the relationships between deposition parameters, defect chemistry and functional performance is still required. Additional efforts are also needed to develop scalable manufacturing routes capable of maintaining high film quality while reducing production costs.

Future research should focus on advanced nanostructuring strategies, interface engineering, heterostructure design and optimization of deposition processes. In gas sensing applications, increasing attention is expected to be devoted to the integration of artificial intelligence and machine learning algorithms for enhanced signal processing, selectivity and real-time data analysis. Nevertheless, the successful implementation of AI- and ML-assisted sensing systems will require the availability of reliable and representative datasets, as well as improved sensor stability and reproducibility. Variations in operating conditions, sensor drift, and cross-sensitivity effects may significantly influence the performance and generalization capability of trained models. Therefore, future efforts should focus not only on algorithm development but also on data quality, standardization, and robust validation procedures to ensure practical deployment in real-world applications. Furthermore, the increasing complexity of sensing environments may require the development of explainable and interpretable machine learning models capable of providing reliable predictions while maintaining transparency in decision-making processes. The combination of advanced Cu_x_O_γ_ sensing materials with robust AI-driven data analysis frameworks may enable the development of adaptive sensing platforms capable of operating under dynamic real-world conditions. However, achieving this goal will require close integration of materials engineering, sensor design, data science, and standardized evaluation methodologies. Such developments may accelerate the implementation of Cu_x_O_γ_ thin films in next-generation sensing and photoelectric technologies.

## Figures and Tables

**Table 1:** Comparison of spin coating and spray pyrolysis techniques used for fabrication of Cu_x_O_γ_ thin films.

Parameter	Spin Coating	Spray Pyrolysis
Deposition principle	Centrifugal spreading of precursor solution followed by drying and annealing	Atomization of precursor solution onto heated substrate followed by thermal decomposition
Typical processing temperature	Room temperature during coating; 200–600 °C during annealing	Typically 250–500 °C during deposition
Film characteristics	Uniform and smooth films; precise thickness control	Morphology tunable from porous to dense; suitable for thicker coatings
Advantages	High thickness uniformity; simple process control	Scalable; suitable for large-area deposition
Limitations	Limited substrate area; material loss during spinning	Droplet-induced defects; morphology control may be challenging
Scalability	Moderate to low	High

**Table 2:** Representative studies on Cu_x_O_γ_ thin films fabricated using PVD-based techniques together with selected deposition parameters and main structure–property relationships.

Deposition Method	Substrate	Process Parameters	Main Findings	Ref.
Direct current (DC) magnetron reactive sputtering	Alumina, sapphire (0001)	Cu target 99.99%; Ar/O_2_ total flow 25 sccm; O_2_ ratios: 10, 20, 30, 40%; 32 W; ~6.5 × 10^−3^ Torr; room temperature (RT); 20 min; target–substrate distance 10 cm	Phase changed from Cu/Cu_2_O at 10% O_2_, through Cu_4_O_3_ at 20–30% O_2_, to single-phase CuO at 40% O_2_. Thickness decreased from 320 to 200 nm with increasing O_2_. CuO film showed CO response of 127% at 375 °C for 91 ppm CO.	[74]
DC reactive magnetron sputtering	Soda–lime glass	Cu target 99.999%; base pressure ~10^−7^ Torr; Ar/O_2_ total flow 30 sccm; pressure 5–15 mTorr; power 50 W; deposition 30 min; substrate rotation 45 rpm; substrate heating 150, 250, 350 °C; post-annealing in N_2_ at 250 or 450 °C for 30 min	Band gap was tunable from 1.30 to 2.04 eV. Optimized RT sputtering at 10 mTorr and O_2_/Ar = 8/22 standard cubic centimeters per minute (sccm) followed by 250 °C annealing gave mobility 34.2 cm^2^ V^−1^ s^−1^. Heated deposition at 150 °C gave mobility 113.7 cm^2^ V^−1^ s^−1^.	[75]
DC reactive magnetron sputtering	Cu substrate	DC power: 90 W; pressure: 6.3 × 10^−3^ Torr; O_2_/Ar ratios: 1:1 and 1:2; substrate temperatures: RT and 300 °C	Optical absorptance and thermal emittance strongly depended on oxygen concentration and substrate temperature. Variations in Cu^+^/Cu^2+^ ratio significantly affected optical response and solar selective behavior.	[76]
Reactive DC magnetron sputtering + vacuum annealing	Borosilicate glass	Cu target 99.9%; Ar/O_2_ = 9/6 sccm; 300 K; 3 × 10^−3^ mbar; 1.4 W cm^−2^; film thickness 250 ± 5 nm; vacuum annealing at 5 × 10^−6^ mbar, 623 or 700 K, 1 h	As-deposited films were CuO. Vacuum annealing at 623 K produced mixed CuO/Cu_2_O, while 700 K produced single-phase Cu_2_O. Grain size increased from 12.8 to 58.4 nm. Transmittance at 600 nm increased from ~15% to 72%.	[72]
DC sputtering + annealing	Glass	Cu target; Ar atmosphere; 30 W; deposition times: 10, 20, 30 min; thicknesses: 60, 120, 180 nm; annealing at 450 °C for 1 h	Film thickness strongly affected optical properties and photocatalytic activity. The 120 nm film gave the highest H_2_ production, 24 μmol. The 60 nm film showed the best CO_2_ photoreduction toward CH_2_O, while the 120 nm film produced CH_3_OH and CH_2_O.	[77]
Radio frequency (RF) sputtering + annealing	Si; glass only for additional comparison	Stoichiometric CuO target 99.99%; Ar atmosphere; Low gas pressure 3 mTorr and high gas pressure 100 mTorr; RT deposition; calcination at 600 °C for 1 min in N_2_; annealing 300–700 °C	Growth pressure and annealing changed crystallinity, roughness and oxidation state. Higher annealing temperatures increased peak intensity/sharpness, improved crystalline quality, increased crystallite size, and reduced strain/stress. Minor Cu_2_O contribution appeared after high-temperature annealing.	[78]
RF magnetron sputtering	Glass	RF power 200 W; O_2_ flow 2 sccm; variable sputtering pressure: 2, 3, 6 and 12 mTorr; RT deposition; annealing at 150 and 250 °C for 2 h	Pressure strongly affected phase formation. At 3 mTorr, strong Cu_2_O (111) was observed. At 6 mTorr, polycrystalline Cu_2_O with (111)/(200) planes formed. Band gap of the 3 mTorr film was 2.75 eV. Surface roughness increased from 2.14 nm at 3 mTorr to 3.94 nm at 6 mTorr.	[79]
PVD Cu deposition + thermal oxidation	glass	Cu evaporation; Cu film ~300 nm; vacuum 5 × 10^−5^ mbar; source–substrate distance 16 cm; air oxidation at 250–400 °C for 60 min; heating ramp 15 °C/min	Cu_2_O was favored below 300 °C, while CuO formed above 300 °C. Band gap varied from 1.54 to 2.21 eV. Carrier concentration increased from 1.61 × 10^12^ to 6.8 × 10^12^ cm^−3^. Resistivity decreased from 5.4 × 10^6^ to 2.4 × 10^5^ Ω cm.	[80]
RF magnetron sputtering	Si	Cu target 99.99%; RF power 250 W; O_2_/Ar ratio 1:7; pressure 2 Pa; substrate temperature 300–400 °C; target–substrate distance 8 cm	Optimal films were obtained at 350 °C. They showed monoclinic CuO with predominant (111) orientation, crystallite size ~24 nm, roughness ~45 nm, band gap 1.68 ± 0.01 eV and conductivity 0.4 S cm^−1^. Areal capacitance reached 387 mF cm^−2^ with 95% retention after 1000 cycles.	[81]
RF magnetron sputtering	Si, glass, stainless steel, carbon	Cu target; Ar/O_2_ atmosphere; total gas flow 50 sccm; O_2_ flow 2–6 sccm; O_2_ concentration 4–12%; pressure 0.5, 1 or 1.5 Pa; power density 2.3 W cm^−2^; substrate unheated or 350 °C; target horizontal or tilted by 23°; target–substrate distance 8–12 cm	Oxygen flow strongly controlled composition and conductivity. CuO films with conductivity 9.3 × 10^−1^ S cm^−1^ were obtained at 12% O_2_. Pressure, target–substrate distance, temperature and target orientation affected morphology, texture and columnar growth.	[82]

**Table 3:** Representative copper precursors used for ALD of Cu_x_O_γ_ thin films.

Precursor Family	Representative Precursor	Advantages	Limitations	Typical Oxide Phase	Ref.
β-diketonates	Cu(acac)_2_	High stability, well-established chemistry	Relatively low volatility, higher deposition temperature	CuO, Cu_2_O	[103,105]
Aminoalkoxides	Cu(dmap)_2_	Good reactivity and growth rate	Narrow process window	Cu_2_O, CuO	[106]
Amidinates	[Cu(sBuAMD)]_2_	Higher volatility, lower deposition temperatures	More complex synthesis	Cu_2_O, CuO	[107]
β-diketonates	Cu(hfac)_2_	High volatility, good vapor transport properties	High volatility, good vapor transport properties	Cu_2_O	[108]

**Table 4:** Representative ALD-based studies on Cu_x_O_γ_ thin films, including deposition conditions and selected physicochemical properties.

Deposition Method	Substrate	Process Parameters	Main Findings	Ref.
Atmospheric pressure spatial atomic layer deposition (AP-SALD)	Borosilicate glass	Copper(I) hexafluoroacetylacetonate vinyltrimethylsilane (Cu(I)(hfac)(tmvs)) precursor; precursor temperature: 65 °C; line temperature: 70 °C; substrate temperature: 125–260 °C; co-reactants: N_2_ (Cu), H_2_O (Cu_2_O), O_3_ (CuO)	Selective deposition of Cu, Cu_2_O and CuO thin films achieved by changing only the co-reactant; phase purity confirmed by XRD, Raman, XPS and X-ray absorption spectroscopy (XAS); CuO films obtained without post-annealing.	[109]
Atmospheric pressure spatial atomic layer deposition (SALD)	ZnO-coated substrates/photodiode structures	Cu(acac)_2_ and O_3_ precursors; deposition temperature: 175–275 °C; growth rate increased from 0.05 Å/cycle (175 °C) to 0.35 Å/cycle (275 °C)	Amorphous, pinhole-free ultrathin CuO films obtained; XPS and XRD confirmed pure CuO (tenorite phase); CuO/ZnO heterojunction photodiodes exhibited rectifying behavior and fast UV response.	[110]
Plasma-enhanced ALD (PE-ALD)	Si and Si/SiO_2_ wafers	Bis(N,N’-di-sec-butylacetamidinato)dicopper(I) [Cu(sBu-amd)]_2_ precursor; substrate temperature: 150 °C; precursor source temperature: 110 °C; H_2_ and O_2_ plasma used as co-reactants; H and O radical ratios of 1:0, 3:1 and 0:1 used to obtain Cu, Cu_2_O and CuO phases, respectively; post-deposition annealing at 350 °C in air or 600 °C under vacuum	Tunable Cu, Cu_2_O and CuO phases obtained by varying H_2_/O_2_ plasma ratio; self-limiting growth behavior confirmed; vacuum annealing increased grain size and reduced copper oxidation state; p-type thin film transistors (TFTs) exhibited on/off current ratio of ~10^5^.	[111]
Thermal ALD	Si substrates; trench structures	Copper(II) acetate monohydrate (Cu(OAc)_2_) and H_2_O precursors; deposition temperature: 180–220 °C; precursor source temperature: 175–185 °C; growth rate: ~0.11–0.13 Å/cycle; saturation achieved using 2 s precursor pulses	Phase-pure, polycrystalline and impurity-free Cu_2_O thin films deposited; conformal coating with 100% conformality achieved on trench structures; Cu_2_O photoconductor devices exhibited response in visible wavelength range.	[112]
Low-temperature thermal ALD	Si(100) and soda–lime glass	Bis(dimethylamino-2-propoxy)copper(II) (Cu(dmap)_2_) and O_3_ precursors; deposition temperature window: 80–140 °C; reactor pressure: ~5 mbar; precursor sublimation temperature: 65 °C; growth rate: 0.2–0.3 Å/cycle	Polycrystalline monoclinic CuO thin films deposited at low temperatures; self-limiting ALD growth observed; relatively low impurity concentration reported; linear dependence between film thickness and number of ALD cycles confirmed.	[106]
Thermal ALD	Planar and three-dimensional SiO_2_/Si substrates	Cu(dmap)_2_ and O_3_ precursors; deposition temperature: ~140 °C; reactor pressure: 200–260 Pa; cycle sequence: 2-2-2-5 s (Cu precursor pulse–purge–O_3_ pulse–purge)	CuO thin films successfully deposited on planar and 3D substrates; XRD confirmed CuO phase formation; resistivity measurements revealed a resistivity drop below 4 K.	[104]
Thermal ALD	Si(100) with native SiO_2_	Dehydrated Cu(hfac)_2_ and diethylzinc (DEZ) precursors; substrate temperature: 190 °C; precursor temperatures: 80 °C (Cu(hfac)_2_) and 5 °C (DEZ); 650 ALD cycles; Cu(hfac)_2_ pulse: 300 ms; DEZ pulse: 50 ms; Ar purge: 5 min; growth-per-cycle: ~0.7 Å/cycle	In situ Time-of-Flight Mass Spectrometry (TOFMS) revealed volatile surface reaction by-products and confirmed simultaneous full and partial ligand exchange mechanisms during Cu ALD; XAS confirmed predominantly metallic Cu films (~85 at.%) with Zn contamination ≤1 at.% and only trace Cu oxide formation after air exposure; dense and conformal Cu thin films with ~45 nm thickness were obtained.	[113]
PE-ALD	Si and quartz substrates	Cu(dmap)_2_ precursor with O_2_ plasma; deposition temperature window: 90–150 °C; self-limiting growth behavior observed; precursor decomposition reported at 180 °C.	Polycrystalline CuO thin films with low surface roughness were deposited at temperatures as low as 90 °C; optoelectronic properties strongly depended on deposition temperature, including band gap (1.08–1.16 eV), work function (4.82–5.15 eV) and valence band maximum (5.33–5.48 eV)	[114]

**Table 5:** Representative CuO-based thin film gas sensors reported within the last five years: deposition methods, structural/physicochemical properties of active layers and sensing performance toward various target gases.

No.	Gas	CuO Deposition Method	Structural/Physicochemical Properties of Active Layer	Gas Sensing Performance	Ref.
1.	H_2_	DC magnetron sputtering	Monoclinic CuO; nanocrystalline grains; Pd-enhanced surface activity; thickness: 283–967 nm.	Optimum temperature: 300 °C; response: 3 (1000 ppm); response/recovery: 10 s/50 s; high H_2_ selectivity.	[133]
2.		Sol–gel spin coating	Porous nugget-like morphology; reduced crystallite size after Pd doping; improved conductivity.	Detection: 1000–15,000 ppm; operating temperature reduced to ~60 °C after Pd doping; ~4× higher response.	[134]
3.		Reactive magnetron sputtering	Cu/Cu_2_O/TiO_2_ nanocrystalline phases; porous morphology; heterojunction-related carrier modulation.	Detection: 100–1000 ppm; best response for (Cu0.56Ti0.44)Ox; low-temperature operation.	[135]
4.	CO_2_	SILAR	Ba-doped monoclinic CuO; preferred (−111) orientation; nanoporous morphology; particle size reduced from ~140 to ~30 nm after Ba doping; increased surface roughness; p-type behavior; optical band gap: 1.59–1.75 eV.	Highest response for CuO:6%Ba; response: 9.4% at RT and 82.2% at 150 °C; response/recovery: 5.6 s/5.44 s; high selectivity toward CO_2_ over NO, CO and NH_3_; good repeatability and long-term stability.	[136]
5.		Sol–gel dip coating	Polycrystalline monoclinic CuO; preferred (111) and ($\bar{1}$11) orientations; porous nanocrystalline morphology; thickness: 189–279 nm; crystallite size: 24–41 nm; optical band gap: 1.47–1.52 eV; p-type conductivity; increased porosity and hydrophobicity for thicker films.	Highest response (~34%) obtained for the thickest porous film at RT (10,000 ppm CO_2_); enhanced sensing associated with increased porosity, grain size and charge transport; improved gas diffusion and adsorption capability.	[137]
6.		Thermal oxidation of patterned Cu thin films followed by Au nanoparticle functionalization	Thermally oxidized CuO thin films; pure CuO phase confirmed by Raman spectroscopy; compact morphology with dispersed Au nanoparticles (~20 nm); Au functionalization increased active adsorption sites and surface reactivity.	Pristine CuO response: ~32% at 350 °C and 2000 ppm CO_2_; Au-functionalized CuO response increased up to 365% at 300 °C; nearly linear response with CO_2_ concentration; response/recovery: 4.3/4.4 min; ~13-fold sensitivity enhancement after Au functionalization.	[138]
7.		Modified sol–gel method combined with spin coating	Spin-coated CuO thin films (~240 nm); pure monoclinic CuO phase; porous micro-/mesoporous morphology; crystallite size: ~32 nm; high concentration of adsorbed oxygen species, oxygen vacancies and hydroxyl groups; high surface reactivity toward CO_2_ adsorption.	Response: ~21% for 8354 ppm CO_2_ at 300 °C and up to ~114% for 39 300 ppm CO_2_; effective operation at 250–300 °C; fast response (~5 s); high selectivity toward CO_2_ over CO and NOx; sensing attributed to formation of carbonate/hydroxyl carbonate surface species.	[139]
8.	NO_2_	Cold-wall CVD	Structural/physicochemical properties: CVD-grown monoclinic CuO thin films; grain size: 86–128 nm; RMS roughness: 7–15 nm; thickness: 134–152 nm; lower-temperature deposition produced smaller grains, higher roughness and increased density of dangling oxygen bonds.	Highly selective NO_2_ sensing at 200 °C; detection limit: 300 ppb; maximum response: 279.1% (sample A) and 220.92% (sample B); sensing strongly dependent on grain size, roughness and surface defect density; improved adsorption and charge transfer for low-temperature-grown films.	[140]
9.		Spray pyrolysis	Monoclinic CuO thin films with crystallinity increasing with precursor concentration; average crystallite size ~13 nm; net-like rough morphology observed for 0.15 M films providing increased active surface area and adsorption sites; optical band gap values of ~2.6–2.9 eV; p-type semiconducting behavior.	Highest NO_2_ response (~56%) obtained for 0.15 M film at 200 °C (100 ppm NO_2_); detection limit: 5 ppm; response/recovery: ~20.57 s/~3.92 min; selective response toward NO_2_ over other oxidizing and reducing gases; enhanced sensing associated with rough, porous morphology and increased adsorption sites.	[141]
10.		Spin coating of CuO nanoparticles (prepared by pulsed laser ablation)	Spin-coated CuO/BSi nanocrystalline thin films; preferential (111)/(−111) orientation; crystallite size: 17–25 nm; porous stone-like morphology after 2 h annealing; slight band gap reduction (1.73→1.71 eV); homogeneous elemental distribution.	Highest NO_2_ sensitivity (~90%) obtained after 2 h annealing at room temperature (~38 °C); response/recovery: ~3.78 s/~1.04 s; enhanced sensing attributed to increased porosity, larger surface area and improved gas diffusion; prolonged annealing reduced porosity and sensitivity (~4%).	[142]
11.	CO	Reactive DC magnetron sputtering	Au:CuO nanoplasmonic thin films with Au nanoparticles (~107 nm) semi-embedded in CuO matrix; optical transparency and stabilization of Au nanoparticles; APTES functionalization improved gas adsorption and modified surface roughness/refractive index.	Room-temperature optical LSPR-based CO sensing (50 ppm); APTES-functionalized films showed enhanced sensitivity; best performance for 0.1% 3-Aminopropyl)triethoxysilane (APTES) solution with wavelength shift of ~0.163 nm and ~3× higher sensitivity than unmodified films; enhanced sensing attributed to interactions between CO molecules and amino groups of APTES.	[143]
12.		Modified sol–gel spin coating	Polycrystalline monoclinic CuO thin films with porous nanostructured morphology; thickness-dependent microstructure; increasing thickness affected grain growth, porosity, carrier transport and gas diffusion pathways.	CO sensing strongly depended on film thickness; optimized thickness improved sensitivity and response/recovery behavior; thinner films promoted faster gas diffusion, while thicker films provided larger adsorption volume and enhanced CO interaction with CuO surface; sensing associated with modulation of hole concentration in p-type CuO.	[144]
13.	NH_3_	Spray pyrolysis	Monoclinic CuO; porous, rough morphology; nanocrystalline structure; p-type semiconducting behavior; high density of adsorption sites.	Enhanced NH_3_ sensing with good selectivity and repeatability; sensing improved by porous morphology and increased surface activity toward ammonia adsorption.	[145]
14.	H_2_S	Hybrid PVD approach: DC sputtering of Sn followed by thermal evaporation of Cu	SnO_2_:CuO sandwich-type heterostructure; CuO/SnO_2_ p–n heterojunctions; porous nanograin morphology; enhanced charge transfer and H_2_S adsorption capability.	Exceptional H_2_S sensing response (~22,000 for 500 ppm H_2_S at 200 °C); response/recovery: ~1/6 min; high selectivity over NH_3_, Cl_2_ and C_2_H_4_; excellent repeatability and long-term stability over 150 days.	[146]
15.	HCHO	Inkjet printing	Polycrystalline monoclinic CuO thin films with porous morphology and interconnected nanograins; uniform printed sensing layer with high surface area and abundant active adsorption sites; good film homogeneity and adhesion.	Highly sensitive formaldehyde sensing; detection limit as low as 50 ppb; good selectivity and repeatability; enhanced sensing attributed to porous morphology and efficient gas adsorption/desorption kinetics.	[147]

**Table 6:** Representative examples of Cu_x_O_γ_-based materials and thin film structures investigated for photoelectric, optoelectronic, photoelectrochemical, and photocatalytic applications.

Application Area	Device/Structure	Role of Cu_x_O_γ_ Materials	Key Findings	Ref.
Photodetectors	CuO thin film photodetector deposited on FTO substrate	Photoactive semiconducting layer responsible for light absorption and photocurrent generation	Broad visible light photoresponse, low dark current, stable and reproducible photocurrent generation under illumination	[151]
Photodetectors	Bi-doped CuO thin film/n-Si heterojunction photodetector	CuO thin film acted as the photoactive semiconducting layer responsible for light absorption and photocurrent generation	Bi doping improved photocurrent, responsivity, detectivity, and photosensitivity under illumination compared to non-doped CuO films	[152]
Photodetectors	CuO thin film visible light photodetector	CuO thin film served as the visible light-absorbing photoactive semiconducting layer	Engineering of the CuO grain structure significantly improved responsivity, detectivity, and response speed; the optimized device exhibited responsivity of 15.3 A/W and detectivity of 1.08 × 10^11^ Jones	[4]
Visible/UV photodetectors	p-CuO/n-ZnO heterojunction pyro-phototronic photodetector	CuO acted as the p-type narrow-band-gap semiconducting layer forming a heterojunction with n-ZnO; CuO deposition parameters controlled carrier transport, interface quality, crystallinity, and pyro-phototronic response	Magnetron-sputtered CuO/ZnO heterojunctions exhibited strong photoresponse under both 365 nm and 405 nm illumination. Optimization of CuO sputtering conditions (120 W, 15 min, O_2_/Ar = 1:3) significantly improved responsivity and detectivity. The pyro-phototronic effect enhanced photocurrent and responsivity by more than an order of magnitude compared with the conventional photovoltaic response alone.	[153]
Visible/UV photodetectors	Cu/CuO/Ag MSM photodetector	Self-assembled monoclinic CuO thin films acted as the p-type photoactive semiconducting layer responsible for visible and UV light absorption, carrier generation, oxygen adsorption/desorption processes, and photocurrent modulation	Room-temperature wet chemically synthesized CuO thin films exhibited photoresponse under both UV and visible illumination. The optimized CuO1 M sample showed the best crystallinity, low series resistance (~1.3 Ω), responsivity of ~1.65 µA/W, ON/OFF ratio of ~69, and detectivity of 22 × 10^5^ Jones at only 0.01 V bias.	[154]
Photocatalysis/environmental remediation	CuO–Cu_2_O heterojunction thin film photocatalyst	CuO and Cu_2_O formed a visible light-active heterojunction photocatalyst in which the interface promoted charge separation, reduced electron–hole recombination, improved optical absorption, and enhanced interfacial charge transfer during photocatalytic degradation of organic pollutants	CuO–Cu_2_O thin films exhibited significantly improved visible light photocatalytic degradation of methylene blue compared with individual CuO and Cu_2_O films. The heterojunction structure enhanced IPCE response, reduced charge transfer resistance, and improved photocorrosion stability. The degradation rate constant reached ~0.0241 min^−1^ and the photocatalyst maintained good stability over five cycles.	[12]
Photocatalysis/optoelectronic materials	Spin-coated CuO thin films	CuO thin films acted as visible light-absorbing semiconducting photocatalytic layers	Polycrystalline monoclinic CuO thin films exhibited high optical absorption, narrow band gap (1.63–1.72 eV), characteristic PL emission in the UV–Vis range, and enhanced visible light-driven photocatalytic degradation of organic dyes	[155]
Photocatalysis/environmental remediation	Zn-doped CuO thin films deposited by reactive magnetron sputtering	CuO thin films acted as visible light-active photocatalytic layers, while Zn doping improved charge transfer and reduced electron–hole recombination	Zn doping significantly enhanced the visible light-driven photocatalytic degradation of methylene blue. The optimized Zn-doped CuO film achieved ~82% degradation efficiency and improved photocatalytic stability over five cycles due to reduced charge-transfer resistance and suppressed carrier recombination	[156]
Photoelectrochemical water splitting/hydrogen generation	FTO/CuO/CuBi_2_O_4_ heterojunction photocathode	CuO acted as the visible light-absorbing electron-generating photocathode layer, while the CuBi_2_O_4_ overlayer improved charge transport and reduced photocorrosion	The CuO/CuBi_2_O_4_ heterojunction exhibited enhanced PEC water-splitting performance, improved stability, and photocurrent density up to 1.23 mA/cm^2^ due to reduced carrier recombination and improved interfacial charge transfer	[157]
Optoelectronic photodevices/photodetectors	Ni- and Co-co-doped CuO thin film photodevice	CuO thin films acted as photoactive semiconducting layers for light absorption and photocurrent generation, while Ni/Co co-doping enhanced optoelectronic response	Ni- and Co-co-doped CuO thin films exhibited enhanced responsivity, external quantum efficiency, and detectivity. The CuO(1%)(1%) device showed responsivity of 0.43 A/W, EQE of 100%, and detectivity of 9.55 × 10^9^ Jones, indicating improved optoelectronic performance after co-doping	[158]
Semitransparent photovoltaics/solar harvesters	AP-SALD-grown Cu_2_O/ZnO semitransparent solar harvester	Cu_2_O acted as a p-type visible light-absorbing semiconductor layer in the Cu_2_O/ZnO heterojunction responsible for charge generation and photovoltaic conversion	Open-air AP-SALD-grown Cu_2_O thin films exhibited high hole mobility up to 92 cm^2^ V^−1^ s^−1^ and enabled fabrication of semitransparent Cu_2_O/ZnO solar harvesters with competitive photovoltaic performance despite ultrathin absorber layers	[159]

## Data Availability

No new data were created or analyzed in this study. Data sharing is not applicable to this article.

## Abbreviations

The following abbreviations are used in this manuscript:

ALD	Atomic Layer Deposition
APS-ALD	Atmospheric Pressure Spatial Atomic Layer Deposition
APTES	(3-Aminopropyl)triethoxysilane
BOM	Bill of Materials
CAGR	Compound Annual Growth Rate
CVD	Chemical Vapor Deposition
CBD	Chemical Bath Deposition
DC	Direct Current
DEZ	Diethylzinc
EDX	Energy-Dispersive X-ray Spectroscopy
EQE	External Quantum Efficiency
FTIR	Fourier Transform Infrared
HVAC	Heating, Ventilation, and Air Conditioning
IPCE	Incident Photon-to-Current Efficiency
LSPR	Localized Surface Plasmon Resonance
MFC	Mass Flow Controller
NDIR	Non-Dispersive Infrared
PL	Photoluminescence
PPM	Parts per million
RF	Radio frequency
RMS	Root Mean Square
RPM	Revolutions per minute
RT	Room Temperature
SCCM	Standard Cubic Centimeters per Minute
SEM	Scanning Electron Microscopy
SILAR	Successive Ionic Layer Adsorption and Reaction
TOFMS	Time-of-Flight Mass Spectrometry
UV	Ultraviolet
XAS	X-ray Absorption Spectroscopy
XPS	X-ray Photoelectron Spectroscopy
